# Superspreading, overdispersion and their implications in the SARS-CoV-2 (COVID-19) pandemic: a systematic review and meta-analysis of the literature

**DOI:** 10.1186/s12889-023-15915-1

**Published:** 2023-05-30

**Authors:** Oliver Wegehaupt, Akira Endo, Anna Vassall

**Affiliations:** 1 Institute for Immunodeficiency, Center for Chronic Immunodeficiency (CCI), Medical Center, Faculty of Medicine, University of Freiburg, Breisacherstr. 115, Freiburg, 79106 Germany; 2 Clinic of Pediatric Hematology, Oncology and Stem Cell Transplantation, Medical Center, Faculty of Medicine, University of Freiburg, Freiburg, Germany; 3 Department of Global Health and Development, London School of Hygiene and Tropical Medicine, London, WC1E 7HT UK; 4 Department of Infectious Disease Epidemiology, London School of Hygiene & Tropical Medicine, London, WC1E 7HT UK; 5 The Centre for Mathematical Modelling of Infectious Diseases, London School of Hygiene & Tropical Medicine, London, WC1E 7HT UK; 6 School of Tropical Medicine and Global Health, Nagasaki University, Nagasaki, Japan; 7 Department of Global Health, The Academic Medical Center (AMC), The University of Amsterdam, Meibergdreef 9, 1105 AZ Amsterdam, The Netherlands

**Keywords:** SARS-CoV-2, COVID-19, Superspreading, Overdispersion, Secondary transmission, Transmission pattern, Heterogeneity

## Abstract

**Background:**

A recurrent feature of infectious diseases is the observation that different individuals show different levels of secondary transmission. This inter-individual variation in transmission potential is often quantified by the dispersion parameter k. Low values of k indicate a high degree of variability and a greater probability of superspreading events. Understanding k for COVID-19 across contexts can assist policy makers prepare for future pandemics.

**Methods:**

A literature search following a systematic approach was carried out in PubMed, Embase, Web of Science, Cochrane Library, medRxiv, bioRxiv and arXiv to identify publications containing epidemiological findings on superspreading in COVID-19. Study characteristics, epidemiological data, including estimates for k and R0, and public health recommendations were extracted from relevant records.

**Results:**

The literature search yielded 28 peer-reviewed studies. The mean k estimates ranged from 0.04 to 2.97. Among the 28 studies, 93% reported mean k estimates lower than one, which is considered as marked heterogeneity in inter-individual transmission potential. Recommended control measures were specifically aimed at preventing superspreading events. The combination of forward and backward contact tracing, timely confirmation of cases, rapid case isolation, vaccination and preventive measures were suggested as important components to suppress superspreading.

**Conclusions:**

Superspreading events were a major feature in the pandemic of SARS-CoV-2. On the one hand, this made outbreaks potentially more explosive but on the other hand also more responsive to public health interventions. Going forward, understanding k is critical for tailoring public health measures to high-risk groups and settings where superspreading events occur.

**Supplementary Information:**

The online version contains supplementary material available at 10.1186/s12889-023-15915-1.

## Background

Since the emergence of the novel coronavirus SARS-CoV-2 in late 2019 and the declaration of a public health emergency of international concern by the WHO on 30^th^ January 2020 [1], more than 670 million cases have been recorded as of February 2023 [2]. Numerous efforts have been made to mitigate onward transmission. Knowledge about dispersion characteristics are indispensable for public health policy as it allows tailored control measures, and understanding dispersion may be critical for future pandemics.

The dispersion parameter k is an estimate of the dispersion in the number of secondary transmissions generated by each case. It is critical to estimating the probability of superspreading events in which certain individuals infect unusually large numbers of secondary cases [3]. Lloyd-Smith et al. first described that the distribution of individual transmission potential around the basic reproduction number R0 is frequently right skewed or overdispersed [3, 4]. They introduced the dispersion parameter k that indicates the variance in the number of offspring based on a Negative Binomial distribution [3]. Hence, k can be described as the variation in inter-individual transmission potential whereby low values of k represent higher variation and larger probability of superspreading events.

The ‘20/80 rule’ is a rule of thumb that emphasises the level of variance typically observed in infectious disease transmission: it is not uncommon that only about 20% of primary cases cause 80% of onward transmission [5]. In sexually transmitted and vector-borne diseases, studies often indicate the percentage of most infectious primary cases that account for 80% of secondary cases, serving as a surrogate marker for heterogeneity in individual infectiousness [5].

**Fig. 1:**
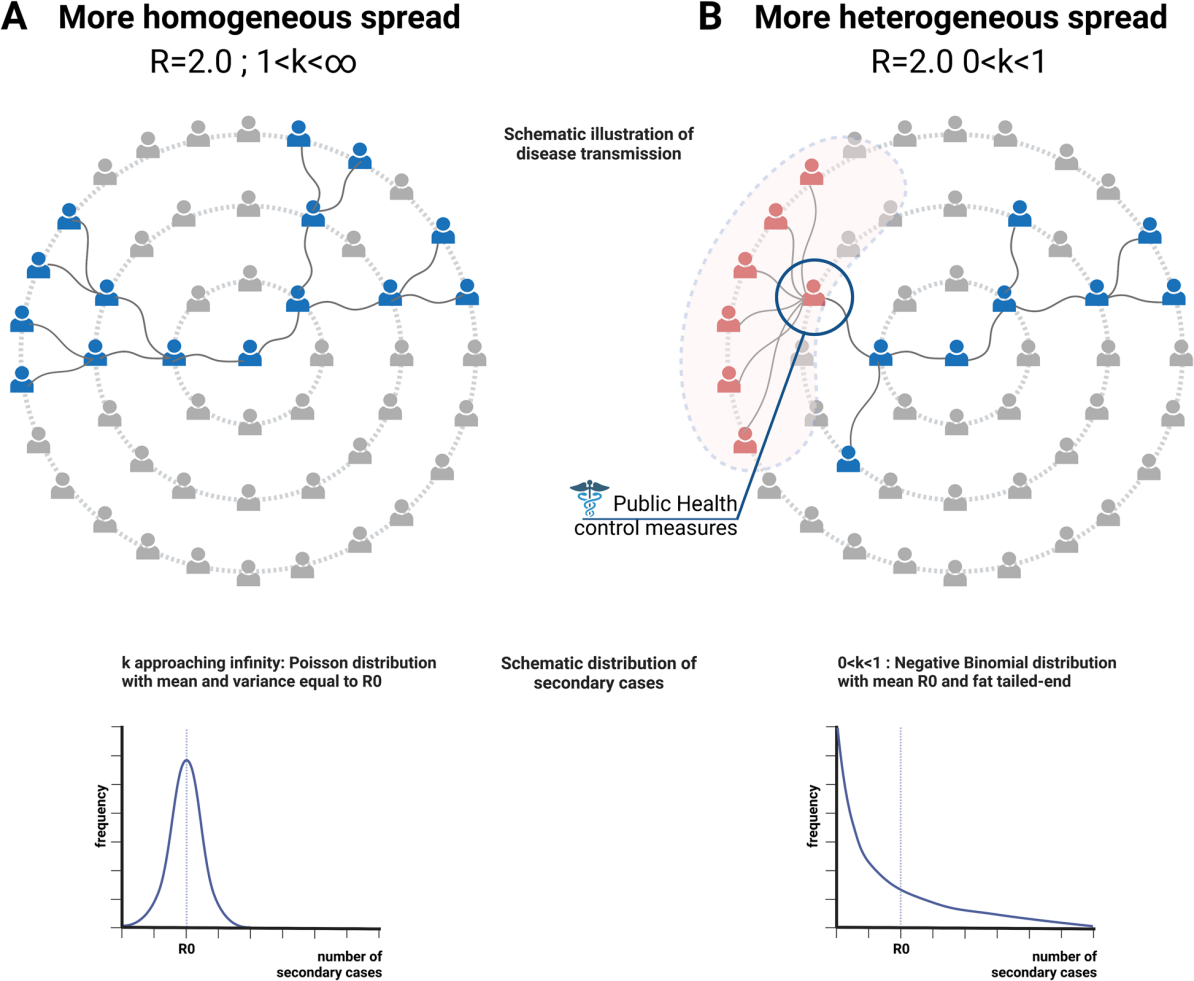
Conceptual framework. Homogeneous and heterogeneous patterns of disease transmission require different control measures. A: Every infected individual passes on the disease to two other people on average, R0 equals two, k approaches infinity, secondary cases show a Poisson distribution with mean and variance equal to R0. As a consequence, Public Health aims at population-wide control measures. B: Infected individuals show different levels of secondary transmission, R0 equals two as in scenario A, but contrastingly k is smaller than one. Secondary cases show a Negative Binomial distribution. Public health measures can target high risk groups or settings where superspreading is likely to occur. Figure created with BioRender.com

If a disease spreads homogeneously, the variance around the base reproduction number R0 is low, k approaches infinity and the distribution of secondary cases approaches Poisson (Fig. 1A). Each case transmits the pathogen onto the next generation rather equally. In this scenario, broad population-wide control measures are necessary and the disease is more difficult to contain [3]. Contrarily, a heterogeneous offspring distribution exhibits a wider dispersion around R0 and k is smaller than one. In this scenario, superspreading events are more likely to occur and become a major concern (Fig. 1B). Large outbreaks happen less frequently but can become more explosive [3]. In this case, by focusing on specific settings or high-risk groups where superspreading occurs, for example large gatherings indoors or people of a certain age group, better containment of virus spread could be achieved without imposing population-wide control measures. Simultaneously, the probability of extinction of the disease is more likely as more cases have no offspring at all.

Where the majority of cases does not contribute to onward transmission, the effective reproduction number could be substantially reduced by preventing superspreading events [6, 7]. In other words, public health interventions that specifically target settings where superspreading events occur could rapidly reduce overall transmission [8]. With high levels of superspreading, individual specific control measures targeting risk groups are likely to outperform population-wide interventions [9].

Infectious diseases with high levels of heterogeneity in transmission, in principle, should be easy to control with public health interventions [10] and this heterogeneity can even be advantageous for control measures [11]. However, this all depends on the ability to effectively identify and reduce transmission related behavior in those populations spreading the disease, without stigmatising those groups, and considering societal equity. Moreover, the effectiveness of measures will also critically depend on R0 and the speed to which any intervention can be implemented.

This study reviews the dispersion parameter k in the context of the SARS-CoV-2 (COVID-19) pandemic, taking into account all-group k estimates as well as values for different subgroups. Following on from this, recommendations for public health are assembled, which studies derived from the calculation of their dispersion parameter estimates. Our aim is to provide a summary that researchers and policy makers can use to better understand the characteristics of k and in doing so can inform future pandemics.

## Methods

### Search strategy and study selection process

A review of the literature was undertaken using a systematic approach. On 4^th^ August 2022 an online search was carried out for publications from 1^st^ January 2020 to 4^th^ August 2022 including the databases PubMed (via NCBI); EMBASE (via OVID); Web of Science; Cochrane Library. As a considerable proportion of work on SARS-CoV-2 / COVID-19 has been published as preprint articles, the “COVID-19 Portfolio” server of the National Institutes of Health (NIH) [12] was additionally searched for non-peer reviewed work filtering for the following databases: MedRxiv; BioRxiv; arXiv.

Three search components were set up for the literature review with the following key concepts: (A) “SARS-CoV-2”, (B) “superspreading” and (C) “dispersion”. Keywords were searched for in all databases (see Table 1). Subject heading searches were conducted in databases where available (in principle PubMed, Embase, Cochrane Library) and where appropriate MeSH terms were identified.

**Table 1:** Search strategy

Steps	Search terms
**Search component A (SARS-CoV-2)**	
1	**Keywords**
	SARS-CoV2 OR SARS-CoV-2 OR Covid19 OR Covid-19 OR "Coronavirus 2019" OR "2019-nCoV" OR "Wuhan coronavirus" OR "Wuhan pneumonia"
2	**Subject headings**
	PubMed: SARS-CoV-2, COVID-19
	Embase: Severe acute respiratory syndrome coronavirus 2, coronavirus disease 2019
3	1 OR 2 (results for search component A)
**Search component B (Superspreading)**	
4	**Keywords**
	Superspread* OR super-spread*
5	**Subject headings**
	Embase: superspreading event
6	4 OR 5 (results for search component B)
**Search component C (Dispersion)**	
7	**Keywords**
	Dispersion OR kappa OR variability OR heterogen* OR "secondary case*" OR "20/80 rule"
8	**Subject headings**
	Embase: Epidemiological data
9	7 AND 8 (results for search component C)
**Inclusion for further assessment**	
10	3 AND 6 AND 9 (Embase)
11	3 AND 4 AND 7 (PubMed)
12	1 and 4 and 7 (Web of Science, Cochrane Library, medRxiv, arXiv, bioRxiv)

For further eligibility of literature the following inclusion criteria had to be met: The study was published on a peer-reviewed or non-peer-reviewed server; the study was based on real world data (e.g. epidemiological surveillance, contact tracing data, genetic analysis of patient samples); the study provided at least one all-group estimate for dispersion parameter k; the study was published between 1^st^ January 2020 and 4^th^ August 2022 in English or German language. Modelling studies were included when they drew on or were validated on epidemiological data.

This study both updated and extended a previously published review [13] in terms of study period covered and data extracted. In doing so, we aimed to expand the understanding of k in SARS-CoV-2 in the rapidly evolving pandemic by including most recent studies, capturing evidence on virus subtypes, but also obtaining k estimates in various subgroups. In addition, we compiled public health recommendations derived from calculated k estimates.

The systematic literature search identified a total of 675 studies (307 on databases for peer-reviewed and 368 on databases for pre-print articles) from 1^st^ January 2020 to 4^th^ August 2022. The Cochrane database search did not retrieve any record. A reference list search of the previous review [13] yielded an additional four studies that met inclusion criteria. 679 records were imported into EndNote software (Version X9 3.3). After removal of 201 duplicates by stepwise deduplication, the remaining 478 records were screened by title and abstract. 434 studies did not meet inclusion criteria (18 not related to SARS-CoV-2; 125 other SARS-CoV-2 related public health topics; 201 insufficient information on dispersion parameters; 69 other natural sciences topic; 18 single case reports; 1 non English/German language; 2 communications) and 44 studies were assessed further for eligibility. Among these, another 16 studies were excluded due to insufficient information on dispersion parameter estimates or pure focus on outbreak simulation. A total of 28 records was finally included in this study: We re-examined and extended data extraction of the 17 studies that were also included in the previous review [13]. Additionally, one major study of 2020 [14] not captured by the previous review was included as well as 10 newly identified and recently published studies (after 10/09/2021) for complete data extraction.

### Quality appraisal, data extraction and synthesis

Quality appraisal of literature was of particular concern in this study as a significant number of non-peer reviewed, and therefore possibly not previously quality checked, COVID-19 work was expected to be eligible for inclusion according to the search strategy. The final set of identified studies was subjected to a critical quality appraisal checklist according to the Critical Appraisal Skills Programme (CASP) guidelines [15] and the quality of cross-sectional studies (AXIS) scale [16]. A set of 13 quality appraisal questions were grouped into the categories “introduction” (2 questions), “methods” (6), “results” (2) and “discussion” (3). The articles were scored based on positive units, ranging from strong (≥10/13 “YES”-units) and good (7-10/13) to weak (<7/13) quality (see supplement A). Emphasis was given to the assessment of the description of methods in order to ensure an a priori valid dispersion parameter calculation.

Articles finally included in this review were first classified by their study characteristics: Author, journal, publication date, title, type of method for estimation of k and type of dataset. Subsequently, epidemiological data was extracted: Estimate of dispersion parameter k; 95% confidence interval (CI) of dispersion parameter k; estimate of basic reproduction number R0; 95% CI of basic reproduction number R0; percentage of cases that is responsible for 80% of secondary cases (20/80 rule); population (size, contacts, clusters); information on analysis of subgroups/ clusters/ settings/ events; study period; and region/ country. Moreover, public health control measures that were recommended based on the identified dispersion characteristics were extracted as well as the type of virus investigated (wildtype vs. variant of concern (VOC)) (see supplement B). To investigate whether the dispersion of secondary cases differs in certain subgroups, available data on k estimates in subgroups was grouped into four categories for further analysis: (1) settings, (2) age of primary case, (3) symptoms at the time of disease transmission, and (4) pre/after public health intervention. Data extracted in this study was primarily used for descriptive and comparative analysis.

### Epidemiological calculations and meta-analysis

For studies that included an all-group mean k estimate, its 95% confidence interval and the number of cases studied (sample size), a meta-analysis was carried out to approximate a pooled global k value for SARS-CoV-2. The analysis included 32 values (obtained from 24 studies). Four studies (containing 8 all-group mean k estimates) were excluded for the pooled analysis because of lack of sample size [6, 17], confidence intervals [10], or both [18]. Two all-group mean k estimates in one study [19] were excluded as upper confidence intervals reached infinity and thus weight in the pooled estimate was considered negligible. The calculation of a global mean k estimate was performed using the inverse variance method for pooling. Hereby, studies containing larger sample sizes and small confidence intervals were given more weight. Obtaining high heterogeneity between values (I^2^ test for heterogeneity=100%), we subsequently employed a random effects model for the measurement of a global mean k estimate. Calculations were carried out in R (version 4.2.3), R-package ‘meta’.

## Results

### Study characteristics

**Fig. 2:**
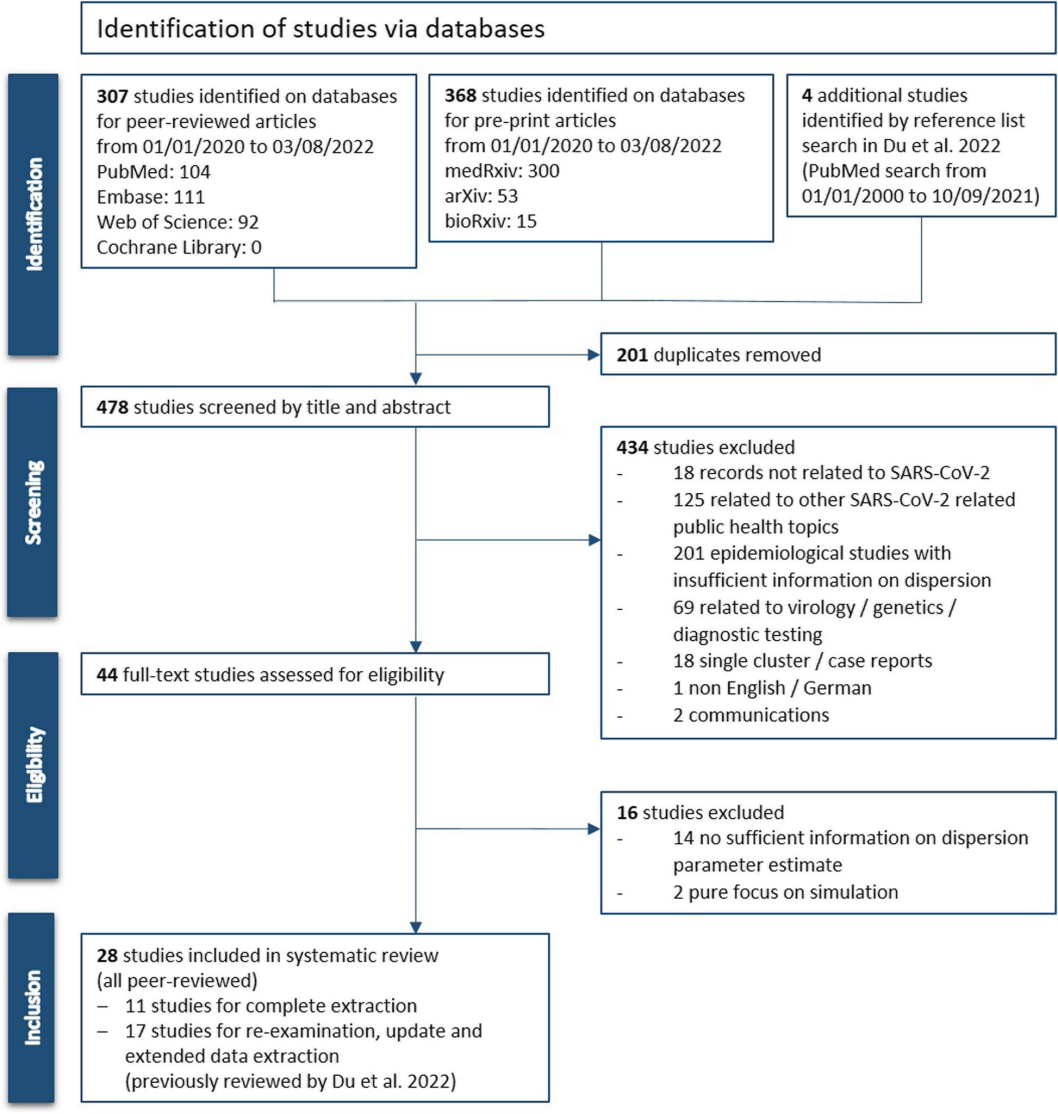
Study selection process.

The PRISMA flowchart shows the detailed study selection process (Fig. 2). Table 2 summarises the study characteristics of included studies, by author in alphabetical order.

**Table 2:** Characteristics of included studies (by author in alphabetical order).

**Nr**	**Author, Journal, Publication Year**	**Title**	**Type of dataset**	**Region**
**1**	Adam et al. [11] Nature Medicine 2020	Clustering and superspreading potential of SARS-CoV-2 infections in Hong Kong	Case line lists and contact tracing data	Hong Kong
**2**	Bi et al. [20] Lancet Infectious Diseases 2020	Epidemiology and transmission of COVID-19 in 391 cases and 1286 of their close contacts in Shenzhen, China: a retrospective cohort study	Surveillance data and contact tracing data	Shenzhen, China
**3**	Endo et al. [7] Wellcome Open Research 2020	Estimating the overdispersion in COVID-19 transmission using outbreak sizes outside China	WHO situation report No 38 (extraction of the number of imported and local cases)	36 countries
**4**	Guo et al. [21] Journal of travel medicine 2022	Superspreading potential of COVID-19 outbreak seeded by Omicron variants of SARS-CoV-2 in Hong Kong	Contact tracing data	Hong Kong
**5**	Gupta et al. [22] PLoS ONE 2022	Contact tracing of COVID-19 in Karnataka, India: Superspreading and determinants of infectiousness and symptomatic infection	Two datasets intersect: daily COVID-19 bulletins and line list of contact tracing data	Karnataka State, India
**6**	Hasan et al. [23] Scientific Report 2020	Superspreading in early transmissions of COVID-19 in Indonesia	Contact tracing data	Jakarta-Depok and Batam, Indonesia
**7**	He et al. [24] BMC Public Health 2020	Low dispersion in the infectiousness of COVID-19 cases implies difficulty in control	Dataset was obtained from Xu et al. (2020) [25]: line list of confirmed case reports. Transmission pair and cluster reconstruction by inferring associations among cases [24]	Mainland China
**8**	James et al. [26] Plos ONE 2021	Model-free estimation of COVID-19 transmission dynamics from a complete outbreak	Anonymised epidemiological data, contact tracing interviews	New Zealand
**9**	Kirkegaard et al. [6] Scientific Reports 2021	Superspreading quantified from bursty epidemic trajectories	Epidemic aggregate data (national surveillance) from the 98 districts in Denmark; total counts of the number of infected (and tested) per day.	Denmark
**10**	Ko et al. [27] International Journal of Infectious Diseases 2022	Secondary transmission of SARS-CoV-2 during the first two waves in Japan: Demographic characteristics and overdispersion	Epidemiological data collected by interviews of confirmed cases (demographic data, clinical information, history of high-risk activities or visit to high-risk venues, contact history)	Japan
**11**	Kremer et al. [28] Scientific Reports 2021	Quantifying superspreading for COVID-19 using Poisson mixture distributions	3 sets of surveillance and contact tracing data of the respective region (Hong Kong: Dataset of Adam et al.; India: dataset of Laxminarayan et al.)	Hong Kong; India; Rwanda
**12**	Kwok et al. [19] J Hosp Infect 2020	Inferring super-spreading from transmission clusters of COVID-19 in Hong Kong, Japan, and Singapore	Public data on confirmed cases, subsequent clustering by epidemiological links (temporal and geographical grouping of one or more index and secondary cases) [19]	Hong Kong; Japan; Singapore
**13**	Lau et al. [18] PNAS 2020	Characterizing superspreading events and age-specific infectiousness of SARS-CoV-2 transmission in Georgia, USA	Surveillance data (including demographic information and geolocation of residence of cases), aggregate mobility data of county inhabitants	Georgia, USA
**14**	Laxminarayan et al. [14] Science 2020	Epidemiology and transmission dynamics of COVID-19 in two Indian states	Surveillance data Contact tracing data	Tamil Nadu and Anda Pradesh State, India
**15**	Lee et al. [29] Int. Journal of Environmental Research and Public Health 2021	Analysis of Superspreading Potential from Transmission Clusters of COVID-19 in South Korea	Epidemiological data; cluster-induced transmissions (“group of cases wherein each case can be associated with the others” [29])	Seoul, South Korea
**16**	Miller et al. [30] Nature Communications 2020	Full genome viral sequences inform patterns of SARS-CoV-2 spread into and within Israel	213 RNA samples from nasopharyngeal swabs (from six major hospitals across Israel)	Israel
**17**	Paireau et al. [8] Eurosurveillance 2022	Early chains of transmission of COVID-19 in France, January to March 2020	Contact tracing data and retrospective epidemiological investigations	France
**18**	Riou et al. [17] BMC medical research methodology 2020	Pattern of early human-to-human transmission of Wuhan 2019 novel coronavirus (2019-nCoV), December 2019 to January 2020	indirect estimate of epidemic size (on 18/01/2020), “based on cases identified in foreign countries before quarantine measures were implemented” [17]	Global
**19**	Ryu et al. [31] Emerg Infect Dis 2022	Serial interval and transmission dynamics during the SARS-CoV-2 Delta variant predominance in South Korea	Contact tracing data	South Korea
**20**	Shi et al. [32] Nat Med 2021	Effective control of SARS-CoV-2 transmission in Wanzhou, China	Epidemiological data and contact tracing data over 4 generations of an outbreak	Wanzhou, China
**21**	Sun et al. [33] Science 2020	Transmission heterogeneities, kinetics, and controllability of SARS-CoV-2	Contact-tracing data	Hunan Province, China
**22**	Tariq et al. [34] BMC Med 2020	Real-time monitoring the transmission potential of COVID-19 in Singapore, March 2020	Surveillance and contact tracing data	Singapore
**23**	Toth et al. [10] PLOS ONE 2021	High variability in transmission of SARS-CoV-2 within households and implications for control	“Serological SARS-CoV-2 antibody test data and prior SARS-CoV-2 test reporting” [10] of households; data paired with maximum likelihood estimate model of importation and transmission	Utah, USA
**24**	Tsang et al. [35] Epidemics 2022	Variability in transmission risk of SARS-CoV-2 in close contact settings: A contact tracing study in Shandong Province, China	Aggregate data of cases and their contacts (contact tracing) Additional surveillance for data of clusters	Shandong Province, China
**25**	Wang et al. [36] Nature Communications 2020	Inference of person-to-person transmission of COVID-19 reveals hidden super-spreading events during the early outbreak phase	208 SARS-CoV-2 genomic sequences with high coverage from China (obtained from GISAID)	China
**26**	Zhang et al. [37] Int. Journal of Environmental Research and Public Health 2020	Evaluating Transmission Heterogeneity and Super-Spreading Event of COVID-19 in a Metropolis of China	Surveillance and contact tracing data	Tianjin, China
**27**	Zhao et al. [9] BMC medical research methodology 2021	Inferencing superspreading potential using zero-truncated negative binomial model: exemplification with COVID-19	Dataset 1-3: Surveillance and contact tracing data of the respective region	Dataset 1: Mainland, China Dataset 2: Hong Kong Dataset 3: Tianjin, China
**28**	Zhao et al. [38] Journal of Travel Medicine 2022	Superspreading potential of SARS-CoV-2 Delta variants under intensive disease control measures in China	Contact tracing data	Guangdong, China

#### Type of articles, date of publication and critical appraisal

All 28 studies finally included were peer-reviewed publications. Any pre-print article that was identified within the study selection process was removed by deduplication as it had meanwhile been published in a peer-reviewed journal. Included studies were published between 2020 and 2022, with the first publication on SARS-CoV-2 superspreading dating to 30^th^ January 2020 [17] and the most recent dating to 11^th^ July 2022 [22]. The quality assessment by critical appraisal revealed high-quality studies (all scoring 10 or higher, see supplement C). The most frequent weakness was the lack of considering limitations in nine publications. The fact that any eligible pre-print article had been published in a peer-reviewed journal in the meantime supported the results of the quality appraisal of included studies.

#### Type of dataset

The included studies performed their calculations using epidemiological data. Five categories of datasets could be identified: the first type of dataset was used by 7 studies and was based on contact tracing data. By asking patients with confirmed SARS-CoV-2 infection to document their close contacts with other infected patients, cases could be placed in a wider transmission network. Calculating the empirical offspring distribution led to an estimate of transmission heterogeneity. A second basis for estimating the dispersion in the population under study was surveillance data. Four studies exclusively used information from surveillance to infer transmission dynamics. In this kind of analysis, temporal and geographical coincidence of one or more index and secondary cases is used as a means to indirectly reconstruct clusters of cases. 13 studies used a combination of surveillance and contact tracing data (see Table 2). We did not observe any significant difference between all-group mean k estimates originating from contact tracing data, surveillance data or a combination of both (see supplement D). Thirdly, two studies drew on SARS-CoV-2 genomic sequences and used phylogenetic trees to deduce dispersion patterns [30, 36]. RNA viruses constantly mutate during replication and transmission. By sequencing the viral genome, epidemiological information can thus be obtained and mapped into transmission networks. As a fourth data source, a study investigating the variability of within household transmission, paired serological SARS-CoV-2 antibody test data with a household survey [10].The fifth type of dataset matched surveillance data (including demographic information and geolocation of the residence of cases) with aggregate high-volume mobility data of the population (obtained by Facebook users who enabled location services on their mobile phones) to infer viral spreading across the region [18].

#### Type of method for estimation of k

In line with a common mode of measuring the heterogeneity of infectiousness and suggested by the pivotal paper of superspreading events in infectious diseases [3], most studies used a negative binomial distribution for the estimation of the dispersion parameter k [6–8, 10, 11, 14, 17–24, 26–29, 31–35, 37, 38]. In addition, one study quantified superspreading potential by using different mixture distributions and compared these to the negative binomial dispersion parameter: the authors suggest a cautious choice of the underlying data generating distribution as the mean in offspring and its variance can become skewed with increasing overdispersion if incorrect assumptions about the type of distribution are made [28]. Finally, two studies analysed genomic SARS-CoV-2 sequences obtained by patients’ samples and subsequently used these for phylodynamic analyses for the estimation of k [30, 36].

#### Countries

**Fig. 3:**
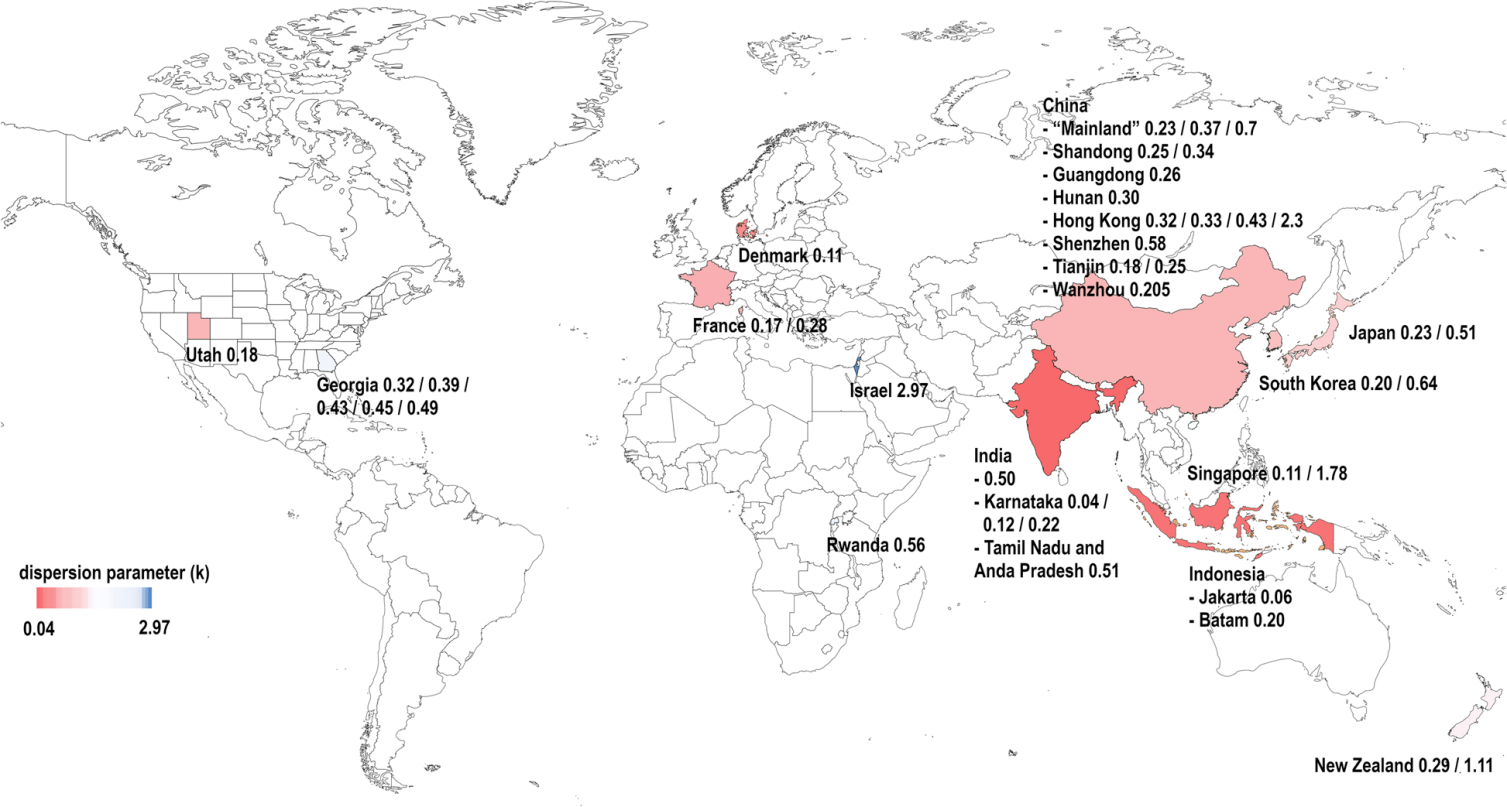
Geographical mapping of reported k estimates. Shown are all-group point estimates of k. Colour-coding based on the countries’ values in the range of k. Created with mapchart.net

Studies investigated SARS-CoV-2 superspreading in 12 countries: China [9, 24, 36] (including specific reports on Hong Kong [9, 11, 19, 21, 28], Shenzhen [20], Tianjin [9, 37], Wanzhou [32] and the provinces of Hunan [33], Guangdong [38] and Shandong [35]), Denmark [6], France [8], India (regions of Karnataka [22], Tamil Nadu and Anda Pradesh [14, 28]), Israel [30], Indonesia [23] (Jakarta Depok, region of Batam), Japan [19, 27], New Zealand [26], Rwanda [28], Singapore [19, 34], South Korea [29, 31], and the United States of America (states of Georgia [18] and Utah [10]) (Fig. 3). Two studies examined patterns of SARS-CoV-2 transmission from a global perspective [7, 17].

### Epidemiological parameters

#### Estimates for dispersion parameter k

**Fig. 4:**
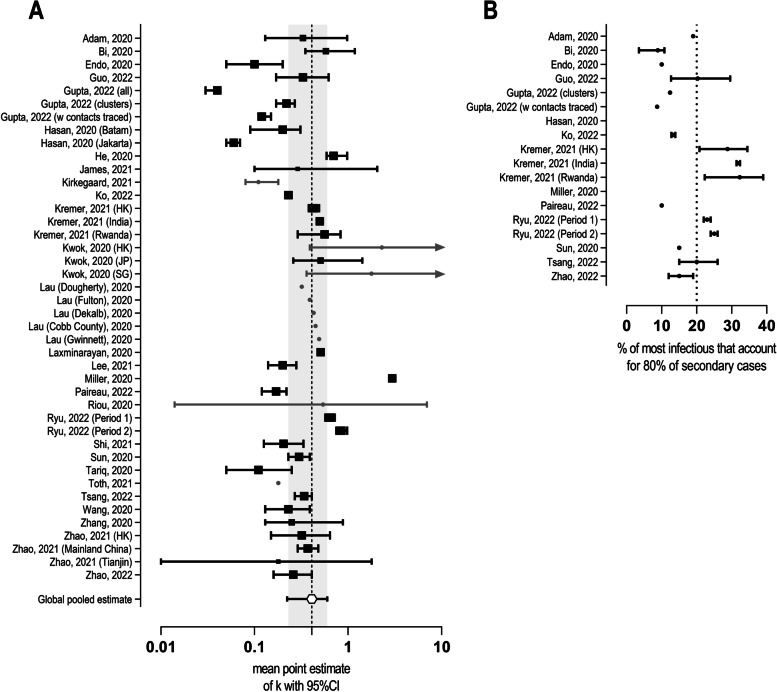
All-group point estimates of k and proportion of primary cases accounting for 80% of onward transmission. (**A**) All-group point estimates of k with 95% CI arranged in alphabetical order. Dashed line indicates mean, area in grey indicates 95% CI of global pooled estimate. Arrows indicate that upper 95% confidence interval reaches infinity. Point estimates in grey are not included in meta-analysis for global pooled estimate. **B** Proportion of most infectious primary cases that generate 80% of secondary casesarranged in alphabetical order. Dashed line indicates empirical ‘20/80 rule’.

**Fig. 5:**
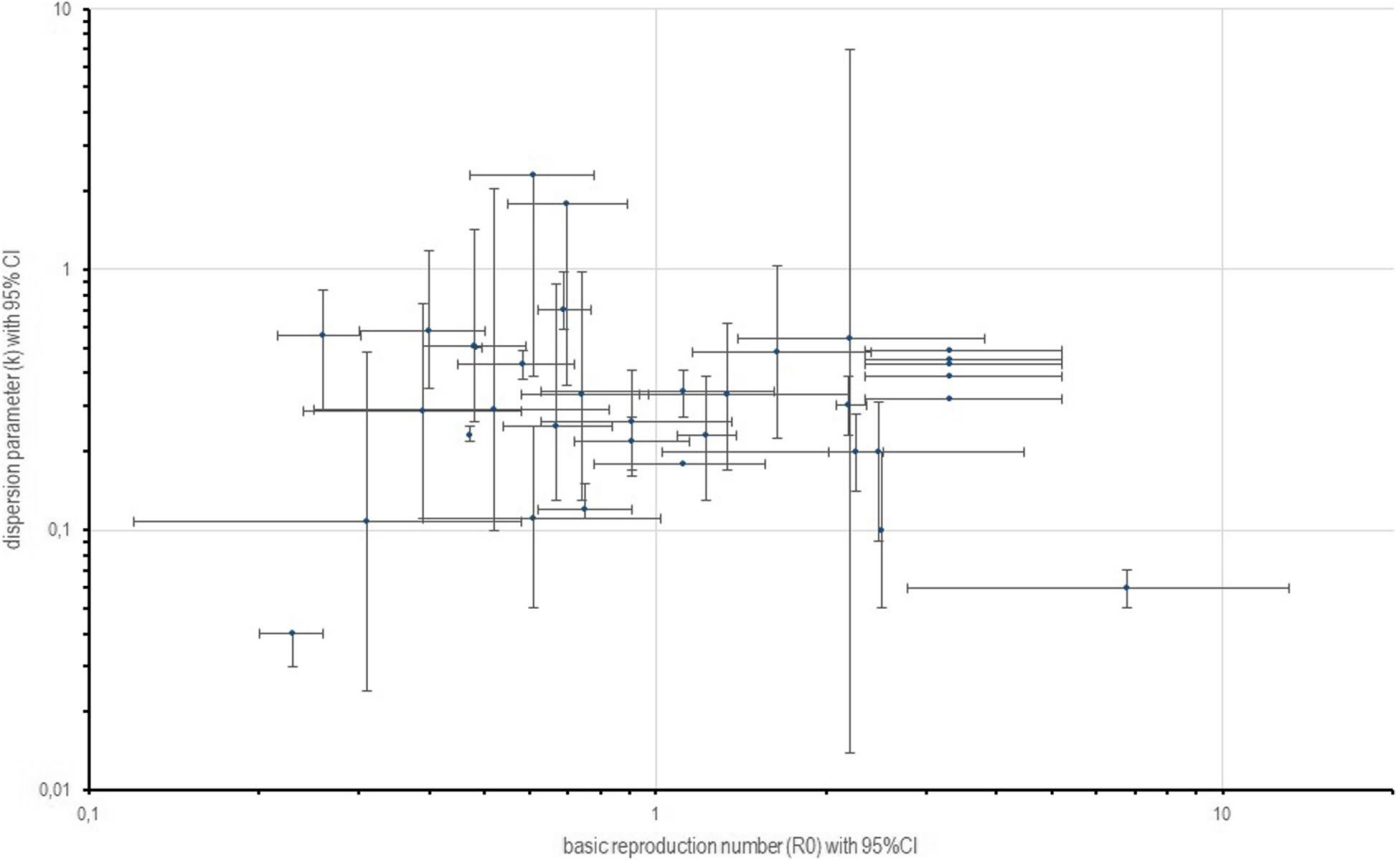
R0 and corresponding k values. Shown are extracted R0 and corresponding k values (with 95% CI). Upper CI limit not depicted if reaching infinity.

All studies provided a point estimate and 95% CI for the dispersion parameter k, indicating the extent of heterogeneity in disease transmission. 93 % of studies (26 of 28) reported mean k estimates lower than one and found a high degree of superspreading potential. Mean k estimates ranged from 0.04 (0.03, 0.04) [22] to 2.97 (2.86, 3.08) [30]. The median of reported mean k point estimates was 0.31. In total, 42 all-group point estimates of k were reported across 28 studies. Employing a weighted meta-analysis of 32 point estimates (of 24 studies), the global pooled mean estimate of k was 0.41 (0.23, 0.60). Figure 4A illustrates the all-group mean k estimates for all studies and the global pooled mean estimate. Table 3 shows all epidemiological data extracted from 28 publications. Paired estimates of R0 and k for each study are displayed in Fig. 5.

**Table 3:** Data on epidemiological parameters. WT: Wildtype. VOC: Variant of concern. N/D: no data

**Nr**	**Author, Year**	**Estimate for** k **(95% CI)**	**Estimate for** R0 **(95% CI)**	**20/80 rule (95% CI)**	**Study population**	**Information on subgroups / clusters / events**	**Study period**	**Region**	**Virus strain WT/ VOC**
**1**	Adam, 2020 [11]	0.43 (0.29, 0.67) [observed offspring distribution and negative binomial distribution] 0.33 (0.13, 0.98) [additional likelihood model based on all local clusters]	0.58 (0.45, 0.72) [observed offspring distribution and negative binomial distribution] 0.74 (0.58, 0.97) [add. likelihood model based on all local clusters]	19% Note: 69% of primary cases did not generate secondary cases	1,038 cases 533 cases linked to 1 of 137 clusters	N/D	23/01/2020 – 28/04/2020	Hong Kong	WT
**2**	Bi, 2020 [20]	0.58 (0.35, 1.18)	0.4 (0.3, 0.5)	8.9% (3.5, 10.8%)	391 cases (1,286 contacts)	N/D	14/01/2020 – 12/02/2020	Shenzhen, China	WT
**3**	Endo, 2020 [7]	Estimate for R0 set to 2.5:0.1 (0.05, 0.2) Joint estimation of R and k: 95%CI: (0.04, 0.2)	k point estimate for R0=2.5 Joint estimation of R and k: 95% CI: (1.4, 12)	~10%	2,788 cases	N/D	Up to 27/02/2020	36 countries	WT
**4**	Guo, 2022 [21]	0.33 (0.17, 0.62)	1.34 (0.94, 2.19)	20.3% (12.7, 29.6%)	234 cases (2 clusters)	N/D	02/01/2022 – 21/01/2022	Hong Kong	Omicron BA.1 + BA.2
**5**	Gupta, 2022 [22]	0.04 (0.03, 0.04).	0.23 (0.20, 0.26)		6824 cases (all cases)		09/03/2020 – 13/06/2020	Karnataka state, India	WT
		0.12 (0.11, 0.15))	0.75 (0.62, 0.91)	8.7% Note: 79.4% of primary cases did not generate secondary cases	956 cases (those with contacts confirmed to have been traced)	Asymptomatic: *n*=753 R=0.41 (0.32, 0.52) k=0.12 (0.09–0.15) Symptomatic: *n*=203 R=2.04 (1.56–2.67) k=0.29 (0.23, 0.37)	09/03/2020 – 13/06/2020		
		0.22 (0.17, 0.27)	0.91 (0.72, 1.15)	12.4% Note: 71.2% of primary cases did not generate secondary cases	3 largest clusters: 394 cases (#1: 221 cases, #2: 97 cases, #3: 76 cases)	Bellary cluster: *n*=221 R=1.04 (0.76, 1.40) k=0.23 (0.17, 0.30) Delhi convention cluster: *n*=97 R=0.84 (0.54, 1.25) k=0.34 (0.22-0.48) Pharmaceutical company cluster: *n*=76 R=0.80 (0.48, 1.29) k=0.32 (0.20-0.46)	09/03/2020 – 21/07/2020		
**6**	Hasan, 2020 [23]	Jakarta-Depok 0.06 (0.05, 0.07) Batam 0.20 (0.09, 0.31)	Jakarta-Depok 6.79 (2.79, 13.07) Batam 2.47 (1.03, 4.48)	10–15%	Jakarta-Depok 1,199 Batam 89 cases	N/D	Jakarta-Depok 02/03/2020 – 31/03/2020 Batam 19/03/2020 – 07/04/2020	Jakarta-Depok and Batam region, Indonesia	WT
**7**	He, 2020 [24]	0.70 (0.59, 0.98)	0.69 (0.62, 0.77)	N/D	9,120 cases	N/D	15/01/2020 – 29/02/2020	Mainland China	WT
**8**	James, 2021 [26]	All subclinical cases before alert level: 1.11 (0.15-∞) All subclinical cases at alert level: 0.29 (0.10, 2.05)	All subclinical cases before alert level: 0.70 (0.18, 1.40) All subclinical cases at alert level: 0.52 (0.25, 0.83)	“20% of cases among adults responsible for 65–85% of transmission” [26]	1,499 cases (627 domestic cases were assigned to 18 clusters)	Domestic clinical cases before alert level: <10 years: *n*=10 R=0.87 (0.33, 1.38) k=3.17 (0.32, ∞) 10-65 years: *n*=336 R= 1.49 (1.41, 1.56) k=0.70 (0.60, 0.81) >65 years: *n*=36 R=1.51 (1.14, 1.81) k=0.50 (0.30, 0.84) Domestic clinical cases at alert level: <10 years: *n*=23 R=0.25 (0.09, 0.44) k=0.80 (0.13, ∞) 10-65 years: *n*=433 R=0.63 (0.58, 0.67) k=0.41 (0.32, 0.50) >65 years: *n*=65 R=1.27 (1.08, 1.49) k=0.23 (0.15, 0.35)	25/03/2020 – 27/04/2020 Proclaim of alert level: 25/03/2020	New Zealand	WT
**9**	Kirke-gaard, 2021 [6]	0.11 (0.08, 0.18)	For R=1.4, 10% of cases cause 70-87% of all infections	N/D	98 municipalities (surveillance at national level)	N/D	26/02/2020 – 17/11/2020	Denmark	WT
**10**	Ko, 2022 [27]	0.23 (0.22, 0.25)	0.47 (0.45, 0.49)	13.3% (12.8, 13.9%) Note: 76.7% of primary cases did not generate secondary cases	67,761 cases data 16,471 primary cases analysed for secondary transmission	Age: 0-19y *n*=847 R=0.36 (0.30, 0.43) k=0.22 (0.17, 0.29) Age 20-39y *n*=7647 R=0.41 (0.38, 0.43) k=0.21 (0.19, 0.23) Age 40-69y: *n*=5832 R=0.52 (0.49, 0.55) k=0.29 (0.26, 0.32) Age 70+y: *n*=1996 R=0.62 (0.55, 0.69) k=0.21 (0.18, 0.24)	15/01/2020 – 31/08/2020	Japan	WT
**11**	Kremer, 2021 [28]	HK: 0.43 (0.38, 0.49)* India: 0.50 (0.50, 0.51)* Rwanda: 0.56 (0.29, 0.83)*	HK: 0.583 (0.448, 0.718) India: 0.484 (0.480, 0.494) Rwanda: 0.259 (0.216, 0.302)	HK: 28.8% (20.8, 34.5%) India: 31.9% (31.4, 32.4%) Rwanda: 32.3% (22.3, 39.0%)	HK: 1,038 cases India: 84,965 cases (575,071 exposed) Rwanda: 795 cases	N/D	HK: 23/01/2020 – 18/04/2020* India: By 01/08/2020* Rwanda: By 31/12/2020*	Hong Kong (HK) India Rwanda	WT
**12**	Kwok, 2020 [19]	HK: 2.30 (0.39, ∞) / (0.02, 4.58)* JP: 0.51 (0.26, 1.42) / (0.21, 1.59)* SG 1.78 (0.36, ∞) / (0.09, 3.47)*	HK: 0.61 (0.47, 0.78) JP: 0.48 (0.39, 0.59) SG: 0.70 (0.55, 0.89)	N/D	89 (HK), 251 (JP) and 103 (SG) cases These consisted of 35 (HK), 131 (JP), and 31 (SG) clusters of secondary cases	N/D	Up to 03/03/2020	Hong Kong (HK) Japan (JP) Singapore (SG)	WT
**13**	Lau, 2020 [18]	0.45 (Cobb County) 0.43 (Dekalb) 0.39 (Fulton) 0.49 (Gwinnett) 0.32 (Dougherty)	Mean R0: 3.30 (2.34, 5.2)	2% of primary cases generate 20% of total infections	9,559 symptomatic cases	Age group <60 years: 2.78 (2.10, 4.22) times larger average of the mean number of offspring than >60y cases, and tend to produce a more extreme number of offspring	01/03/2020 – 03/05/2020	Georgia, USA	WT
**14**	Laxmina-rayan, 2020 [14]	0.51 (0.49, 0.52)	1.1 to 1.4	N/D Note: 71% of primary cases did not generate secondary cases	84,965 cases (575,071 exposed)	N/D	05/03/2020 – 01/08/2020	Tamil Nadu and Anda Pradesh State, India	WT
**15**	Lee, 2021 [29]	0.20 (0.14, 0.28)	Reff 2.26 (2.02, 2.53)	N/D	3,088 cases (61 clusters) analysed (cluster: >20 cases)	Settings: Religious groups: k= 0.16 (0.06, 0.38) Convalescent home: k= 0.5 (0.11, 0.22) Hospital: k= 0.34 (0.09, 1.27) Workplace and school: k= 0.20 (0.10, 0.42) Leisure facilities: k= 0.23 (0.11, 0.49)	04/03/2020 – 04/12/2020	Seoul, South Korea	WT
**16**	Miller, 2020 [30]	2.97 (2.86, 3.08)*	N/D	5-10%	213 cases	N/D	Up to 22/04/2020	Israel	WT
**17**	Paireau, 2022 [8]	Contact tracing: 0.17 (0.12, 0.22) Retrospective investigation: 0.28 (0.09, 0.47)	mean number of secondary cases identified per index case: 0.3–0.9	Contact tracing: 10% Retrospective investigation: 16%	6,082 contacts of 735 cases were traced; some infectors/ infectees paired by epidemiological investigation	Superspreading events occurrence: work (10 cases), neighbourhood dinner (6 cases), family/religious gathering (10 cases), mixed type settings: family, hospital and co-worker contact (31 cases)	24/01/2020 – 30/03/2020	France	WT
**18**	Riou, 2020 [17]	0.54 90% high density interval (0.014, 6.95)	2.2 90% high density interval (1.4, 3.8)	N/D	stochastic simulations that were consistent with the epidemiological findings at study date	N/D	Up to 18/01/2020	Global	WT
**19**	Ryu, 2022 [31]	Period 1: 0.64 (0.57, 0.72) Period 2: 0.85 (0.75, 0.98)	No exact value depicted	Period 1: 23% (22, 24%) Period 2: 25% (24, 26%)	Period 1: 19,635 cases (2,169 transmission pairs) Period 2: 34,569 cases (3,609 transmission pairs)	N/D	Period 1: 11/07/2021 – 24/07/2021 Period 2: 25/07/2021 – 15/08/2021	South Korea	Delta (B.1.617.2)
**20**	Shi, 2021 [32]	G1: 0.484 (0.226, 1.038) G2: 0.284 (0.110, 0.735) G3: 0.107 (0.024, 0.482) G4: 0.048 (0.004, 0.602) Overall G1-G4: 0.205 (0.126, 0.334)	G1-G2: 1.64 (1.16–2.40) (before control measures) G2-G3: 0.39 (0.24, 0.58) G3-G4: 0.31 (0.12, 0.58)	N/D	183 cases (2,100 total close contacts)	Stratifying by symptoms of infector: G1-G2 asymptomatic: R=2.44 (1.21, 6.75) G1-G2 symptomatic: R=1.63 (1.03, 2.59) G2-G3 asymptomatic: R=0.15 (0.00, 0.35) G2-G3 symptomatic: R=0.54 (0.32, 0.84) G3-G4 asymptomatic: R=0.06 (0.00, 0.21) G3-G4 symptomatic: R=0.62 (0.24, 1.48)	21/01/2020 – 10/04/2020	Wanzhou, China	WT
**21**	Sun, 2020 [33]	0.30 (0.23, 0.39)	2.19 (2.08, 2.36)	15%	1,178 cases (15,648 contacts) (19,227 separate exposure events)	Subset: 870 SARS-CoV-2 cases, 14,622 close contacts (exclusion of cases whose infected contacts reported a travel history to Wuhan): Household contacts: k=0.72 Extended family contacts: k=0.64 Social contacts: k=0.19 Community contacts: k=0.14	16/01/2020 – 03/04/2020	Hunan province, China	WT
**22**	Tariq, 2020 [34]	0.11 (0.05, 0.25)	0.61 (0.39, 1.02)	N/D	247 cases (18 clusters)	N/D	23/01/2020 – 17/03/2020	Singapore	WT
**23**	Toth, 2021 [10]	0.43 (0.02, 2.0) (called d_h_ here, variability of transmission within households); converted to overall k=0.18	1.12 (0.78, 1.56)	N/D	28,321 household members (in 9,224 households)	No subgroups of “household member” analysed	04/05/2020 – 15/08/2020	Utah, USA	WT
**24**	Tsang, 2022 [35]	For all contact settings: 0.34 (0.27, 0.41) For all secondary cases: 0.25 (0.12, 0.37)	1.12 (0.63, 1.62)	20% (15, 26%) Note: 64% (55, 72%) of primary cases did not generate secondary cases	199 cases reported 97 primary cases in 89 close contact groups; 3,158 contacts analysed in 81 clusters.	Setting: Household: k=0.34 (0.25, 0.43) Healthcare facility: k=0.076 (0.05, 0.10) Workplaces: k=0.054 (0.031, 0.076) Air transportation: k=0.014 (0.006, 0.022)	22/01/2020 – 30/03/2020	Shandong Province, China	WT
**25**	Wang, 2020 [36]	0.23 (0.13, 0.39)	1.23 (1.09, 1.39)	N/D	208 cases	10 randomly selected phylogenetic trees: k estimate was higher than for the maximum clade credibility (MCC) tree. This subgroup analysis suggests that the phylogeny tends to underestimate superspreading events, as the MCC is the more accurate method.	24/12/2019 – 14/02/2020*	China	WT
**26**	Zhang, 2020 [37]	0.25 (0.13, 0.88)	0.67 (0.54, 0.84)	N/D	135 cases (43 transmission chains)	Before 01/02/2020 k=0.14 (0.04, 0.63) R= 0.74 (0.39, 1.61) After 01/02/2020 k= 0.77 (0.14, 31.47) R= 0.53 (0.29, 0.96)	21/01/2020 – 26/02/2020	Tianjin, China	WT
**27**	Zhao, 2022 [38]	0.26 (0.16, 0.41)	0.91 (0.63, 1.36)	15% (12, 19%)	126 cases	N/D	05/2021 – 12/2021	Guangdong China	Delta (B.1.617.2)
**28**	Zhao, 2021 [9]	Zero-truncated version: Dataset 1: 0.37 (0.29, 0.48) Dataset 2: 0.32 (0.15, 0.64) Dataset 3: 0.18 (0.01, 1.79)	N/D	N/D	Dataset 1: 2,214 cases (1407 transmission pairs, 807 infectors, 1,241 terminal cases) Dataset 2: 290 cases (169 transmission pairs, 91 infectors, 153 terminal cases) Dataset 3: 47 cases (36 clusters, 7 infectors, 11 terminal cases))	Study compares results to non-truncated version (Xu et al.; Adam et al.; Zhang et al.): k values in zero-truncated framework are lower than those obtained by the non-truncated version for all datasets Otherwise no subgroups analysed	Dataset 1: 15/01/2020 – 29/02/2020 Dataset 2: 23/01/2020 – 28/04/2020 Dataset 3: 21/01/2020 – 26/02/2020	Dataset 1: Mainland, China Dataset 2: Hong Kong Dataset 3: Tianjin, China	WT

^*^ value extracted from Du et al. [13]

#### Proportion of primary cases accounting for 80% of onward transmission

Sixteen studies presented the fraction of most infectious that generate 80% of secondary cases in SARS-CoV-2, ranging from 8.7% to 32.3% (Fig. 4B). Nine studies found that percentages of less than 20% of cases accounted for 80% of onward transmission.

#### Subgroup analysis

##### Analysis by cluster type and setting

**Fig. 6:**
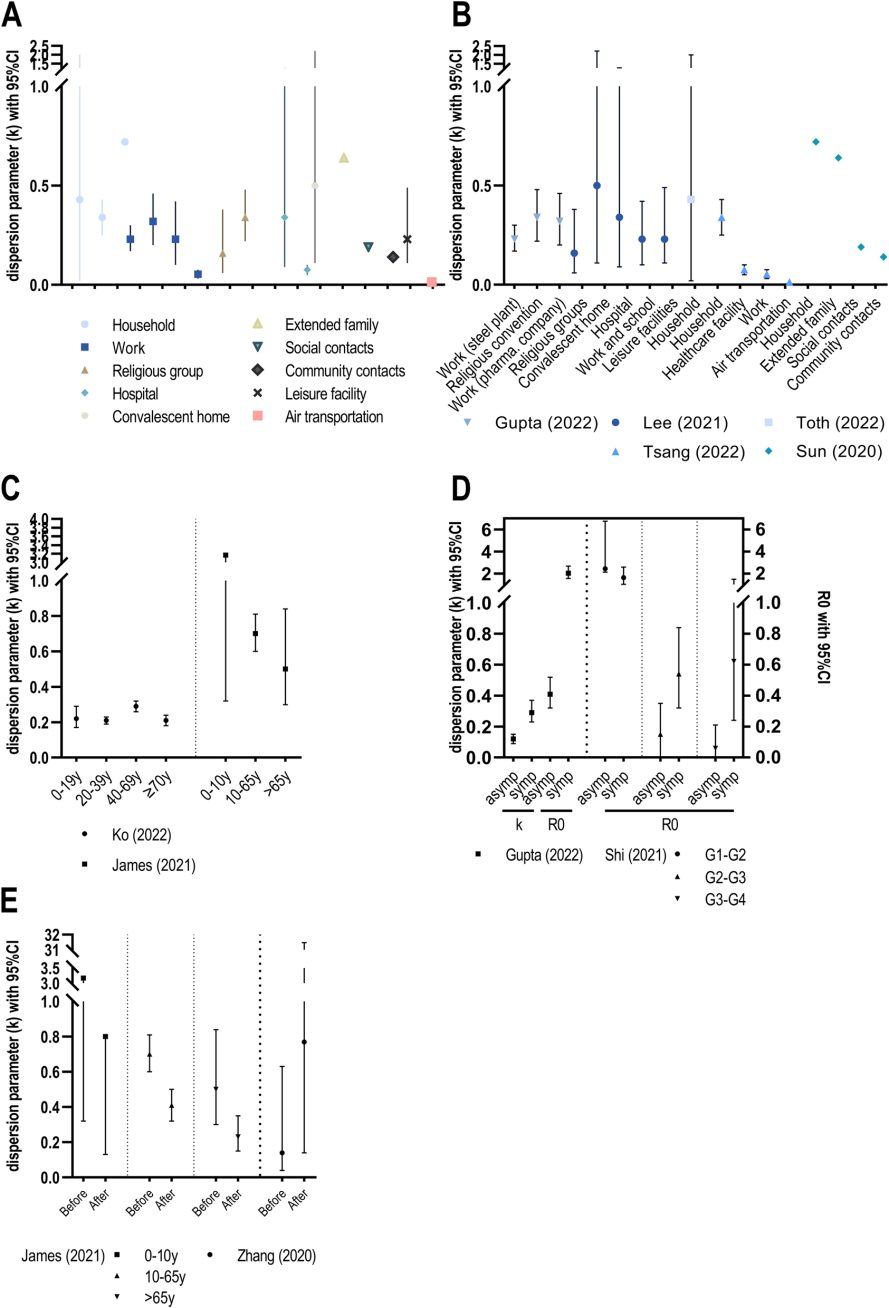
k and R0 estimates for different subgroups. **A** Analysis by cluster type/ setting (by setting). **B** Analysis by cluster type/ setting (by publication). **C** Analysis by age group. **D** Analysis by symptoms. **E** Analysis by public health interventions.

Five studies investigated the dispersion of SARS-CoV-2 infections in specific settings. High levels of overdispersion were present across all settings with mean k estimates ranging between 0.014 and 0.72 (Fig. 6A by setting, Fig. 6B by publication). Three studies identified k estimates in households and four at work with superspreading occurring somewhat less likely in the former than in the latter. Both religious gatherings and hospitals or convalescent homes were identified as risk settings for superspreading [22, 29, 35]. There was an increase in overdispersion and superspreading potential the less close the contacts were [33] (risk in ascending order: household, extended family, social contact and community contact). This result of high overdispersion following sporadic community contacts was consistent with low k values found for leisure facilities [29] and air transportation [35] in two other studies.

##### Analysis by age of infector

A Japanese study found low k estimates for all age groups throughout the entire study period which did not differ significantly. Of note, 80% of primary cases causing secondary transmission belonged to the age group of 20-69 years [27]. The other study stratifying k for age groups reported that children under 10 years played less of a role in the spread of SARS-CoV-2 than adults. With a mean reproduction number of 0.87 (versus 1.49 and 1.51 for adults and elderly people, respectively) and a mean k estimate of 3.17 (versus 0.7 and 0.5 for adults and elderly people, respectively), they generated fewer secondary cases on average and were less likely to be superspreaders [26] (Fig. 6C). Another study divided into two age groups above and below 60 years of age. Though not directly calculating k values, it showed that the average of the mean number of offspring in the age group under 60 years is 2.78 (2.10, 4.22) times larger than in elderly cases and that younger people therefore tended to generate more extreme numbers of offspring [18].

##### Analysis by symptoms

Two studies distinguished between asymptomatic and symptomatic cases. The basic reproduction number was significantly lower in asymptomatic than in symptomatic cases, but overdispersion was higher in the asymptomatic group [22]. Except for the first of transmission generations, a lower R0 in asymptomatic cases was also observed in the other study, which, however, did not determine k values [32] (Fig. 6D).

##### Analysis by public health intervention

Two studies examined whether heterogeneity in individual infectiousness is affected by pandemic control measures. In the first study, after interventions (traffic restriction, quarantine measures) had taken effect, a lower transmission potential and heterogeneity (decrease in R0 and increase in k) was observed [37]. The second study also found a decrease in R0 after alert level introduction (curfews, shutdown of business and schools), but contrarily showed an associated decrease in k across all age groups [26], but if this decrease in k under public health interventions resulted in more superspreading events was not discussed (Fig. 6E).

#### Virus characteristics

Three studies focused on the SARS-CoV-2 variants of concern Delta and Omicron, respectively. Delta is attributed a higher superspreading potential (k=0.26) by the first publication, compared to that of the wild-type in the early pandemic outbreaks [38]. The authors emphasised the risk of superspreading if Delta entered areas with low herd immunity or places where many people meet. The second study analysed the change in transmission dynamics as Delta became the dominant variant in South Korea. A slight increase in k was identified here (0.64 and 0.85 before and at predominance, respectively) [31]. One study looked at heterogeneity in transmission of the Omicron variant and found overdispersed transmission. Compared to Omicron subtype BA.1, the more recent subtype BA.2 has an even greater superspreading potential [21]. The authors hypothesise that the observation of greater susceptibility to superspreading might be explained with low prevalence of vaccination boosters at the time of investigation and only limited natural immunity due to a 'zero COVID-19’ policy in Hong Kong [21].

### Public Health recommendations

Table 4 categorises and lists all public health interventions recommended in the reviewed papers, as stated in the publications, regardless of their feasibility, societal or legal/ human rights implications. The strategy to specifically ban large gatherings and limit capacity in indoor spaces was the most recommended, followed by targeting high-risk groups and large close contact groups. In the category of surveillance and contact tracing, the need for rapid tracing and quarantine for contacts was most frequently suggested. One study that calculated a mean k point estimate also addressed backward tracing as an approach to mitigate viral spread. Population-wide control measures like wearing face-masks and vaccination were also among the recommendations to reduce viral spread despite an overdispersed transmission pattern.

**Table 4:** Public Health recommendations as stated in reviewed publications

**Public Health recommendations**	**Count**	**References**
**Non-pharmaceutical interventions in infected and exposed individuals**		
surveillance and contact tracing	2	[14, 32]
reduce delay from symptom onset to confirmation (testing delay)	2	[22, 32]
rapid isolation of COVID-19 patients	2	[17, 31]
rapid tracing and quarantine for contacts	3	[11, 34, 37]
backward / retrospective tracing	1	[22]
case isolation in dedicated hospitals and contact quarantine in medical observation centers	1	[33]
**Non-pharmaceutical interventions in not necessarily infected or exposed individuals**		
social distancing measures	2	[18, 32]
target the core high-risk groups and large close contact groups	3	[11, 30, 35]
bans on large gatherings and limit capacity in indoor spaces (e.g. target restaurants and entertainment sites, traditional markets, religious gatherings, and weddings)	5	[11, 22, 23, 34, 38]
strengthen non-pharmaceutical intervention capacity	1	[31]
wearing face masks	3	[17, 19, 32]
**Vaccination**	**2**	[31, 35]
**Prevention of international spread**		
prevention of long-distance dissemination, e.g. target flight passengers	1	[35]
border controls	1	[32]

## Discussion

We find here a wide range of work that estimates the heterogeneity in transmission of SARS-CoV-2 and overall, we find consistent evidence of high level of overdispersion across settings. This suggests that public health measures that focus on risk groups may have been effective at slowing transmission, where the disease had not been evenly spreading among the general population.

Heterogeneity in SARS-CoV-2 transmission was present in the early outbreaks of the pandemic as well as in the latest observations and across different variants. Our compilation of k estimates for subgroups classified according to different criteria showed that superspreading occurs across all age groups and in a wide variety of settings. Children may seem to be less heterogeneous transmitters though the number of studies stratifying for age was limited. By contrast, asymptomatic carriers can be particularly hazardous, as they showed more heterogeneous transmission patterns and can thus also contribute to superspreading.

Going forward, a common approach in early pandemic response measures is the so-called backward tracing of cases, recommended by one study [22], in which not only possible contacts of the infected individual are notified, but also the origin of infection is traced back to the index case. This method helps to identify clusters and was largely adopted by Japan in the first wave of infections [27, 39]. Cluster based approaches were shown to be effective in preventing superspreading events and help to terminate transmission chains, where done very promptly [27]. In the case of COVID-19, we found one modelling study comparing backward and forward tracing methods. It suggested that primary cases identified by backward tracing may generate 3-10 times more infections than those identified by forward tracing [40]. The proportion of secondary cases thereby averted was estimated to two to threefold and effectively contributed to outbreak control. These findings are a reminder, that early rapid control efforts can be pivotal even in pathogens with high levels of infectiousness.

Nevertheless, SARS-CoV-2 transmission highlighted the challenge for non-pharmaceutical interventions to specifically target risk groups and settings [11, 30, 37]. As demonstrated in the subgroup analysis, superspreading events occurred in a large variety of settings. In retrospect, heterogeneity of infectiousness was equally present within households and at work. Nevertheless, special attention should probably be paid in pandemic response planning to known indoor and special risk settings (e.g. care facilities, prisons, food processing plants, cruise ships, and large gatherings [41]), as proposed by most of the reviewed studies.

The general observation of overdispersion in SARS-CoV-2 transmission seems to be very robust. Estimates for k were reported across different countries, time points, populations and different viral strains. Moreover, different datasets and methods have been applied for calculations. Together with the presence of study cohorts with large sample sizes, reported estimates of k seemed consistent and plausible overall. However, the dispersion parameter from one region cannot necessarily be transferred to another as populations differ in general composition, immunity level and control measures in place. Interestingly though, the age distribution of a population on a nationwide scale was not likely to be associated with SARS-CoV-2 superspreading potential. In 2020, the median age of reviewed countries ranged from 20.3 years (Rwanda) to 48.2 (Japan) years [42]. The respective k estimates did not correlate with median age across countries.

This study has several limitations. Firstly, the scope of our review did not include direct assessment of the quality of statistical measurements of reviewed publications or the quality of the source datasets. It was also beyond the scope of this work to reconstruct quantities of interest (e.g. for k, R0 or the 20/80 rule) that were not reported in the reviewed studies. These mean that we assumed that all the reported estimates were statistically sound and accurate. Secondly, reported estimates are from datasets collected in various time points in the pandemic under different levels of interventions and/or behavioural changes, which could have deviated the estimates from the “baseline” SARS-CoV-2 dispersion patterns. Moreover, with a growing number of vaccinated individuals from the end of 2020 onward, the virus no longer encounters a fully susceptible population. For these reasons, k estimates would only reflect real-world conditions at the time of investigation. Thirdly, under lockdowns, superspreading events were by default only possible where people were still allowed to meet (e.g. in households or at work). Data on superspreading events in settings that were under restriction (e.g. concert halls, theatres) has been limited. Fourth, being conducted in the middle of a pandemic, the included studies were mostly retrospective and secondary by nature. As the data was primarily collected for other purposes than estimating k (e.g. case isolation), possible estimation approaches were restricted by available data types. Estimates of k may have been more likely to be reported from settings where the collected data was incidentally suitable for estimation, which could be a source of bias. Estimating k is most straightforward when the distribution of the number of secondary transmissions per case is available, e.g. through contact tracing. In such instances, k can be estimated simply by fitting a negative binomial distribution to the observed data. Most of the studies included in our review used this approach and the pooled estimate may have been subject to limitations associated with the data collection, e.g. unidentified epidemiological links. Although some modelling approaches could estimate k from other (less informative) types of data, e.g. cluster sizes [7, 43], they seem to have been rarely used for COVID-19 data, potentially due to data access and technical hurdles. Finally, most studies were conducted before the emergence of variants of concern (VOC); only few studies estimated parameters for VOCs including Delta and Omicron. These variants might have different epidemiological characteristics than wild type SARS-CoV-2. Two of the included studies analysed data containing the Delta variant and only one study covered the Omicron variant, which left the evidence for these variants unestablished.

Taken together, our findings highlight the importance to consider the two key metrics of transmission potential - R0 and k – in parallel in preparing for control measures and to weigh these against each other. There is no “one-fits-all” approach, but in general early indications of overdispersed offspring distribution warrants implementation of targeted measures to mitigate pandemic spread and especially control superspreading events. Approaches for real-time monitoring of transmission heterogeneity and simultaneous estimation of R0 and k from incidence data are currently being explored [44] and should be fully incorporated in surveillance systems.

## Conclusion

In summary, the systematic literature review for superspreading events in the SARS-CoV-2 pandemic with epidemiological characterisation of transmission patterns yielded dispersion parameter estimates that were mostly smaller than one, indicating a high superspreading potential. A combination of forward and backward contact tracing, timely confirmation of cases, rapid case isolation, vaccination, and preventive measures were suggested to be important measures for outbreak control and the suppression of superspreading events. Further investigations have to be performed to analyse new SARS-CoV-2 variants of concern, in particular Omicron subvariants, as data for heterogeneity in transmission is still limited here. Future research will also need to elucidate heterogeneity in transmission in African and Latin American countries for a global picture of dispersion patterns. It should be determined how k is affected in populations of partially vaccinated or recovered people, in particular in remaining susceptibles. Since parts of the population cannot be vaccinated, public health measures will then also have to prevent superspreading in these vulnerable groups.

## Supplementary Information

**Additional file 1: A.** Critical appraisal criteria. **B.** Form for data extraction. **C.** Quality assessment by critical appraisal. **D.** Comparison of all-group mean k estimates by type of dataset.

## Data Availability

All data generated or analysed during this study are included in this published article and its supplementary information files.
