# Supplementary material for: Superspreading, overdispersion and their implications in the SARS-CoV-2 (COVID-19) pandemic: a systematic review and meta-analysis of the literature
Source: BMC Public Health. 2023 May 30;23:1003. doi: 10.1186/s12889-023-15915-1 (PMC10227392; doi:10.1186/s12889-023-15915-1)
Supplement: Supplementary file 1 — Additional file 1: A. Critical appraisal criteria. B. Form for data extraction. C. Quality assessment by critical appraisal. D. Comparison of all-group mean k estimates by type of dataset. [file 12889_2023_15915_MOESM1_ESM.docx]

1. **Critical appraisal criteria**

| Author, Title, Publication date | | Score: | | |
| --- | --- | --- | --- | --- |
|  |  | Yes | No | CT |
| **Introduction** | | | | |
| 1 | Did the article address a clearly focused research question / aim? |  |  |  |
| 2 | Was the aim of the research specific to SARS-CoV-2/ COVID-19? |  |  |  |
| **Methods** | | | | |
| 3 | Was the research design appropriate to address the aims of the research? |  |  |  |
| 4 | Was it clear where and how the source dataset was generated? |  |  |  |
| 5 | Was it clearly defined who the research was about (population)? |  |  |  |
| 6 | Did the authors do enough to assess quality of the included data? |  |  |  |
| 7 | Were the transmission parameters measured appropriate to the aims of the study? |  |  |  |
| 8 | Was the data analysis sufficiently rigorous in its description? |  |  |  |
| **Results** | | | | |
| 9 | Is there a clear and comprehensible presentation of findings? |  |  |  |
| 10 | Were the results internally consistent? |  |  |  |
| **Discussion** | | | | |
| 11 | Were the authors’ discussions and conclusions justified by the results? |  |  |  |
| 12 | Have limitations of the findings been considered? |  |  |  |
| 13 | Is the article potentially valuable to derive policy recommendations for the mitigation of SARS-CoV-2 superspreading events? |  |  |  |

Y: YES, N: NO, CT: Cannot tell

Critical appraisal checklist derived from the Critical Appraisal Skills Programme (CASP) checklists for systematic reviews and qualitative studies; and from the quality of cross-sectional studies (AXIS) scale.

1. **Form for data extraction**

| **No** | **Item** |
| --- | --- |
|  | |
| **Study characteristics** | |
| 1 | Author |
| 2 | Journal |
| 3 | Publication date |
| 4 | Title |
| 5 | Type of method for estimation of *kappa* |
| 6 | Type of dataset |
|  |  |
| **Epidemiological data** | |
| 7 | Estimate of dispersion parameter (*kappa*) |
| 8 | 95% confidence interval (CI) of dispersion parameter (*kappa*) |
| 9 | Estimate of basic reproduction number *R0* |
| 10 | 95% confidence interval (CI) of basic reproduction number *R0* |
| 11 | Percentage of cases that is responsible for 80% of secondary cases (20/80 rule) |
| 12 | Population (size, contacts, clusters) |
| 13 | Information on analysis of subgroups / clusters / settings / events |
| 14 | Study period |
| 15 | Region / Country |
|  | |
| **Virus characteristics** | |
| 16 | Wildtype vs. variant of concern (VOC) |
|  |  |
| **Public Health** | |
| 17 | Control measure recommendations |
|  |  |
| **General** | |
| 18 | Notes / Comments |

1. **Quality assessment by critical appraisal**

| **Author** | **Introduction** | | **Methods** | | | | | | **Results** | | **Discussion** | | | **Score** |
| --- | --- | --- | --- | --- | --- | --- | --- | --- | --- | --- | --- | --- | --- | --- |
|  | **1** | **2** | **3** | **4** | **5** | **6** | **7** | **8** | **9** | **10** | **11** | **12** | **13** |  |
| Adam, 2020 | Y | Y | Y | Y | Y | Y | Y | Y | Y | Y | Y | Y | Y | 13 |
| Bi, 2020 | Y | Y | Y | Y | Y | Y | Y | Y | Y | Y | Y | Y | Y | 13 |
| Endo, 2020 | Y | Y | Y | Y | Y | Y | CT | Y | Y | Y | Y | Y | Y | 12 |
| Guo, 2022 | Y | Y | Y | Y | Y | Y | Y | Y | Y | Y | Y | N | Y | 12 |
| Gupta, 2022 | Y | Y | Y | Y | Y | Y | Y | Y | Y | Y | Y | Y | Y | 13 |
| Hasan, 2020 | Y | Y | Y | Y | Y | Y | Y | Y | Y | Y | Y | N | Y | 12 |
| He, 2020 | Y | Y | Y | Y | Y | Y | Y | Y | Y | N | Y | N | Y | 11 |
| James, 2021 | Y | Y | Y | Y | Y | Y | Y | Y | Y | Y | Y | Y | Y | 13 |
| Kirkegaard, 2021 | Y | Y | Y | Y | N | CT | Y | Y | Y | Y | Y | N | Y | 10 |
| Ko, 2022 | Y | Y | Y | Y | Y | CT | Y | Y | Y | Y | Y | Y | Y | 12 |
| Kremer, 2021 | Y | Y | Y | N | Y | Y | Y | Y | Y | Y | Y | N | CT | 10 |
| Kwok, 2020 | Y | Y | Y | Y | Y | Y | Y | Y | Y | Y | Y | N | Y | 12 |
| Lau, 2020 | Y | Y | Y | Y | Y | Y | Y | Y | Y | Y | Y | Y | Y | 13 |
| Laxminarayan, 2020 | Y | Y | Y | Y | Y | Y | Y | Y | Y | Y | Y | Y | Y | 13 |
| Lee, 2021 | Y | Y | Y | Y | Y | Y | Y | Y | Y | Y | Y | Y | Y | 13 |
| Miller, 2020 | Y | Y | Y | Y | Y | Y | Y | Y | Y | Y | Y | N | Y | 12 |
| Paireau, 2022 | Y | Y | Y | Y | Y | Y | Y | Y | Y | Y | Y | Y | Y | 13 |
| Riou, 2020 | Y | Y | Y | N | N | Y | Y | Y | Y | Y | Y | Y | Y | 11 |
| Ryu, 2022 | Y | Y | Y | Y | Y | Y | Y | Y | N | Y | Y | Y | Y | 12 |
| Shi, 2021 | Y | Y | Y | Y | Y | Y | Y | Y | Y | Y | Y | Y | Y | 13 |
| Sun, 2020 | Y | Y | Y | Y | Y | Y | Y | Y | Y | Y | Y | Y | Y | 13 |
| Tariq, 2020 | Y | Y | Y | Y | Y | Y | Y | Y | Y | Y | Y | Y | Y | 13 |
| Toth, 2021 | Y | Y | Y | Y | Y | Y | Y | Y | Y | Y | Y | Y | Y | 13 |
| Tsang, 2022 | Y | Y | Y | Y | Y | Y | Y | Y | Y | Y | Y | Y | Y | 13 |
| Wang, 2020 | Y | Y | Y | Y | Y | Y | Y | Y | Y | Y | Y | N | Y | 12 |
| Zhang, 2020 | Y | Y | Y | Y | Y | Y | Y | Y | Y | Y | Y | Y | Y | 13 |
| Zhao, 2021 | Y | Y | Y | Y | Y | Y | N | Y | Y | Y | Y | Y | Y | 12 |
| Zhao, 2022 | Y | Y | Y | Y | Y | Y | Y | Y | Y | Y | Y | N | Y | 12 |

Y: YES, N: NO, CT: Cannot tell

1. **Comparison of all-group mean k estimates by type of dataset**


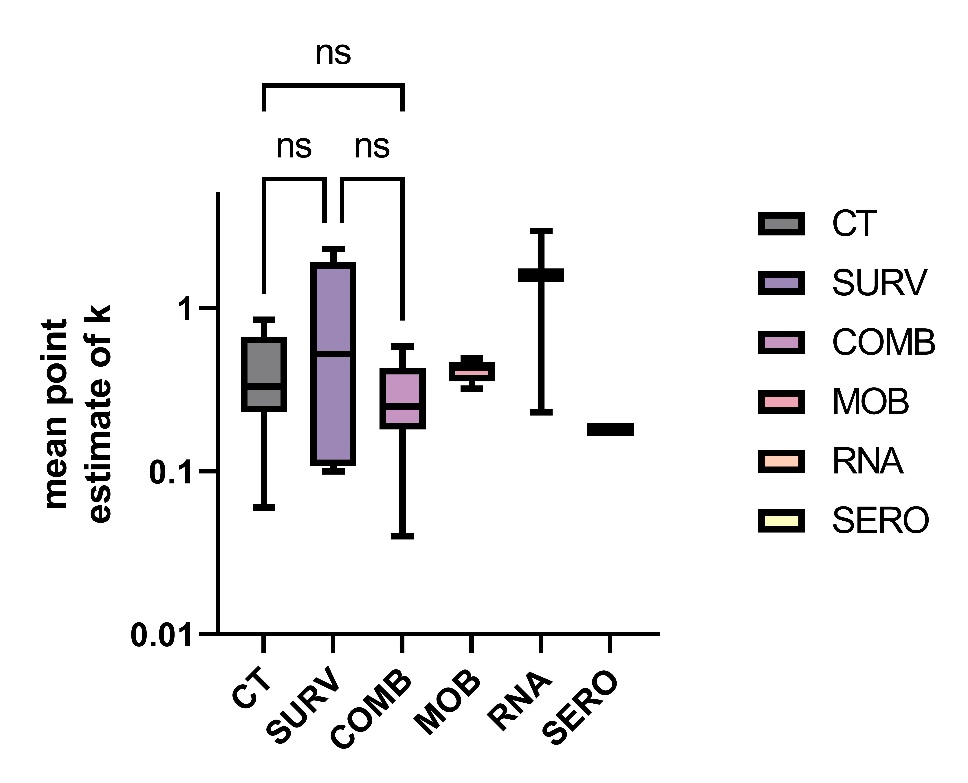


Supplement D: Comparison of all-group mean k estimates by type of dataset. There is no significant difference between values originating from studies using contact tracing data (7 studies), surveillance data (4 studies) or a combination of both (13 studies). Few studies used mobility data (1 study), phylogenetic data from RNA samples (2 studies) or serological data (1 study). Therefore statistical differences of corresponding k estimates were not assessed in these.
CT: Contact tracing data. SURV: Surveillance data. COMB: Combination of contact tracing and surveillance data. MOB: Combination of mobility and surveillance data. RNA: SARS-CoV-2 genomic sequences. SERO: Serological SARS-CoV-2 antibody test data with household survey.
